# Supplementary material for: The causal role of circulating inflammatory markers in osteoporosis: a bidirectional Mendelian randomized study
Source: Front Immunol. 2024 Jul 18;15:1412298. doi: 10.3389/fimmu.2024.1412298 (PMC11291241; doi:10.3389/fimmu.2024.1412298)
Supplement: Supplementary file 1 [file DataSheet_1.zip › supplementary material/CIM-OSTEOPOROSIS /GCST90274788.csv_leave_one_out.pdf]

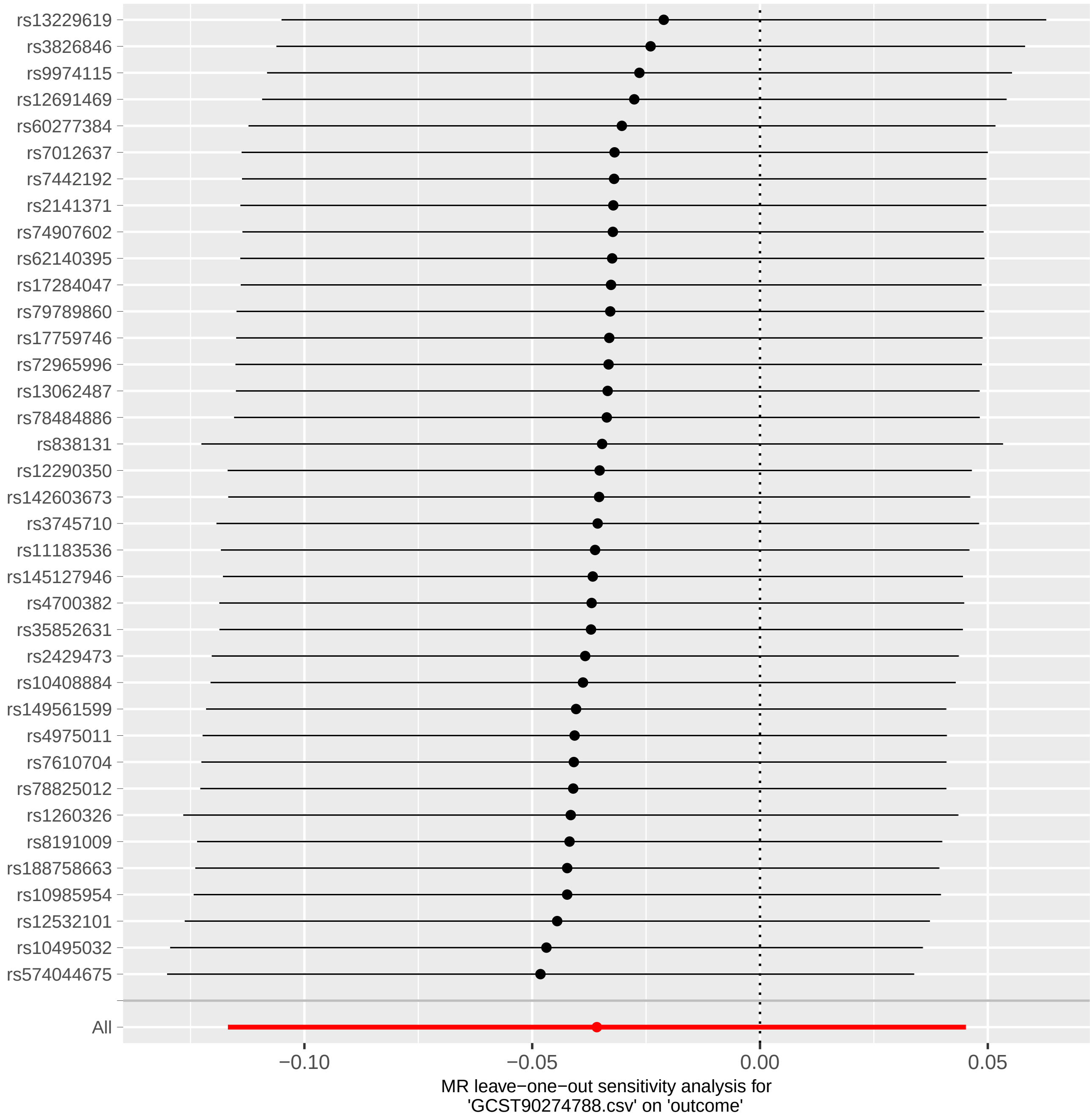
rs13229619
rs3826846
rs9974115
rs12691469
rs60277384
rs7012637
rs7442192
rs2141371
rs74907602
rs62140395
rs17284047
rs79789860
rs17759746
rs72965996
rs13062487
rs78484886
rs838131
rs12290350
rs142603673
rs3745710
rs11183536
rs145127946
rs4700382
rs35852631
rs2429473
rs10408884
rs149561599
rs4975011
rs7610704
rs78825012
rs1260326
rs8191009
rs188758663
rs10985954
rs12532101
rs10495032
rs574044675
All
−0.10
−0.05
0.00
0.05
MR leave−one−out sensitivity analysis for
'GCST90274788.csv' on 'outcome'
